# Lutein nanodisks protect human retinal pigment epithelial cells from UV light-induced damage

**DOI:** 10.3389/fnano.2022.955022

**Published:** 2022-08-15

**Authors:** Anthony Moschetti, Colin A. Fox, Samuel McGowen, Robert O. Ryan

**Affiliations:** Department of Biochemistry and Molecular Biology, University of Nevada Reno, Reno, NV, United States

**Keywords:** lutein, macular degeneration, UV irradiation, nanodisc, retinal pigment epithelial cells

## Abstract

The hydrophobic carotenoid, lutein, was conferred with aqueous solubility upon formulation into reconstituted discoidal high density lipoprotein particles, termed lutein nanodisks (ND). When formulated with phosphatidylcholine (PC), apolipoprotein (apo) A-I and lutein (formulation ratio = 5 mg PC/2 mg apoA-I/1 mg lutein), lutein solubilization efficiency in phosphate buffered saline (PBS) was ~90%. The UV/Vis absorbance maxima for lutein ND in PBS were red shifted by 6–13 nm versus the corresponding lutein absorbance maxima in ethanol. FPLC gel filtration chromatography gave rise to a single major absorbance peak in the size range of ND. Incubation of cultured ARPE-19 cells with lutein ND resulted in lutein uptake, as determined by HPLC analysis of cell extracts. Compared to control incubations, ARPE-19 cells incubated with lutein ND were protected from UV light-induced loss of cell viability. Consistent with this, reactive oxygen species generation, induced by exposure to UV irradiation, was lower in lutein-enriched cells than in control cells. Thus, uptake of ND-associated lutein protects ARPE-19 cells from UV light-induced damage. Taken together, the data indicate ND provide an aqueous lutein delivery vehicle for biotechnological or therapeutic applications.

## Introduction

Age-related macular degeneration (AMD) is the most prevalent cause of vision loss in the developed world, responsible for 8.7% of blindness globally. (Wong et al., 2014). This has been attributed to increased life expectancy as well as tobacco smoke, genetics, oxidative stress, and chronic inflammation. (Seddon et al., 1994; Richer et al., 2004; Zarbin, 2004). The most common form of AMD is the non-exudative (i.e., dry) type, which occurs when retinal pigment epithelial (RPE) cells degenerate, causing secondary photoreceptor cell death and progressive loss of vision. (Del Priore et al., 2002; Liang and Godley, 2003). Currently, there are no treatments available to restore vision to patients with non-exudative AMD.

The Age Related Eye Disease Study demonstrated that nutrient supplementation (vitamin C, vitamin E, ß-carotene, lutein, and zeaxanthin) has a protective effect, slowing both the progression of AMD and the rate of vision loss. (Age-Related Eye Disease Study Research Group, 2001). Subsequently, the Age Related Eye Disease Study 2 revealed that removal of ß-carotene from the supplement formulation reduced rates of lung cancer, while maintaining protection against AMD. (Chew et al., 2014; Musch, 2014). Given that humans are unable to synthesize lutein or zeaxanthin, these compounds must be obtained in the diet. However, lutein is nearly insoluble in aqueous media. (Drugbank online) Since poor aqueous solubility adversely affects oral bioavailability, the efficiency with which orally administered lutein is deposited in the macula is an important consideration.

Consistent with their ability to absorb short-wave blue and UV light, (Bernstein et al., 2001), lutein, zeaxanthin and *meso*-zeaxanthin are the only carotenoids found in the human retina and lens epithelial cells. (Bone et al., 1985; Handelman et al., 1988; Babizhayev, 2011). These xanthophylls protect DNA, lipids and proteins from light-induced damage. (Bian et al., 2012; Silvan et al., 2016). The predominant retinal carotenoid, lutein, (Sommerburg et al., 1998), has been documented to protect spontaneously arising retinal pigment epithelial 19 (ARPE-19) cells from damage caused by prolonged exposure to blue and UV light. (Obana et al., 2008; Aimjongjun et al., 2013). Consistent with this, studies have shown that the concentration of lutein in the macula is inversely correlated with onset, and progression, of AMD. (Seddon et al., 1994; Obana et al., 2008). Despite this correlation, the mechanism by which lutein protects these cells against oxidative stress, or the role lutein deficiency plays in progression of AMD, remain inconclusive. (Kalariya et al., 2008; Obana et al., 2008).

Following intestinal absorption, lutein is transported through the circulatory system as a component of plasma lipoproteins. (Clevidence and Bieri, 1993). Studies have revealed that lutein is transported to the retina as a component of high density lipoproteins (HDL). (Wang et al., 2007; Loane et al., 2010; Mutungi et al., 2010). Thus, it is reasonable to consider that solubilization of lutein into reconstituted HDL (rHDL) particles may be of therapeutic benefit. Previous studies have shown that lutein is conferred with aqueous solubility by incorporation into detergent micelles (Lornejad-Schäfer et al., 2007; O’Sullivan et al., 2004) or liposomes. (Junghans et al., 2001; Pintea et al., 2005; Shafaa et al., 2007). Advantages of discoidal rHDL over micelle and liposomal formulations include the absence of detergents and a uniform nanoscale particle size.

Discoidal rHDL are a structural mimic of naturally occurring HDL species that are biocompatible and non-toxic. (Ryan, 2008). To distinguish bioactive agent-containing particles from classical rHDL, the term nanodisk (ND) is used. (Ryan, 2010). In previous studies, various hydrophobic compounds have been successfully formulated into ND. These include the polyphenol, curcumin, the macrolide polyene antibiotic, amphotericin B and the isoprenoid electron carrier, coenzyme Q_10_. (Oda et al., 2006; Ghosh et al., 2011; Moschetti et al., 2020). Herein, formulation and characterization of lutein ND is described. The results obtained show that lutein ND constitute an aqueous soluble vehicle for lutein delivery to cultured ARPE-19 cells. Compared to control cells, lutein-enriched ARPE-19 cells display improved viability and decreased reactive oxygen species (ROS) generation in response to UV light exposure. Taken together, lutein ND represent a novel lutein delivery vehicle that has the potential to confer protection to photoreceptor cells from light-induced damage associated with AMD.

## Materials and methods

### Materials

Lutein was obtained from Cayman Chemical Company. Fucoxanthin and egg yolk phosphatidylcholine (EYPC) were purchased from Sigma-Aldrich.

### Lutein ND formulation

Lutein ND were formulated using 5 mg EYPC, 2 mg recombinant human apolipoprotein apoA-I (Ryan et al., 2003) and 1.0 mg lutein (from an 8 mg/ml stock solution in tetrahydrofuran) using a variation of the method described by Moschetti et al. (Moschetti et al., 2020) Following bath sonication for ~45 min at 43–45°C the sample clarified, indicating ND particle formation. The sample was then centrifuged at 14,000 × g for 5 min, dialyzed against 20 mM sodium phosphate pH 7.4, 150 mM NaCl (PBS) and stored at 4 °C. When stored in the dark at 4°C in a vessel purged with N_2_ gas, lutein ND are stable for up to one year.

### UV/Vis spectroscopy

Absorbance spectra were collected on a Spectramax M5 instrument using a quartz cuvette. Spectra were obtained for lutein (10 μg) in ethanol, lutein ND in PBS (10 μg as lutein) and control EYPC ND (no lutein) in PBS. Absorbance scans were collected from 350 nm to 570 nm. In solubilization efficiency studies, lutein was added to formulation mixtures comprised of PBS only, apoA-I in PBS, EYPC in PBS or EYPC and apoA-I in PBS. Samples were adjusted to 1.2 ml with PBS and bath sonicated for 45 min at 43–45°C. Following sonication, samples were centrifuged to remove insoluble material and an aliquot (20 μl) of the supernatant transferred to a microfuge tube containing 480 μl 95% ethanol and analyzed by UV/Vis spectroscopy. Lutein content was quantitated using its extinction coefficient in ethanol [145,100 L/(mol cm at 445 nm)] and Beer’s law or by comparison to a standard curve of lutein absorbance in ethanol. (Lornejad-Schäfer et al., 2007). The lutein content of fractions collected following gel filtration chromatography of ND samples was determined in a similar manner.

### ARPE-19 cell culture

ARPE-19 cells were purchased from Biosciences Divisional Services at UC Berkeley. Cells were cultured in DMEM/F-12 (Gibco) media supplemented with 10% fetal bovine serum (Peak Serum), 50 U/ml penicillin, 50 μg/ml streptomycin, 1:100 dilution GlutaMax, and 1:100 dilution sodium pyruvate (Life Technologies, Carlsbad, CA). For cell viability and ROS assays, phenol red-deficient DMEM/F-12 media was used to prevent interference with subsequent colorimetric and fluorescence measurements. Cells were incubated at 37°C in a humidified chamber (5% CO_2_). Cultures were seeded with 1 × 10^6^ cells (100 mm dish), or 4,000 cells/well (96-well plate), and allowed to adhere overnight. The following day, the media was replaced and cells were cultured to 90% confluence over the course of 5 days, with a media change on the 3^rd^ day. Specified incubations were initiated on day 7. UV irradiation studies, cell viability assays, and ROS determination were performed on day 10 after seeding.

### Fast protein liquid chromatography

Lutein ND samples were centrifuged at 15,000 × g for 10 min immediately prior to gel permeation chromatography on a Superdex 200 Increase 10/300 GL column fitted to a GE AKTA Pure fast protein liquid chromatography (FPLC) instrument. Four hundred μl aliquots of lutein ND were applied to the column. Samples were eluted with PBS at a flow rate of 0.75 ml/min. Absorbance was continuously monitored at 280 nm, with collection of 2.0 ml fractions. Fractions corresponding to absorbance peaks were pooled and concentrated to ~500 μl by centrifugal filtration (3,000 Da MWCO). The 280 nm absorbance peaks eluting between 8.2 to 10.2 ml and 12.2–14.2 ml, respectively, were analyzed for phospholipid, lutein, and apoA-I content. EYPC content was determined using a LabAssay Phospholipid kit (Wako Pure Chemical Corp., Japan) according to the manufacturer’s instructions. Lutein content was measured spectroscopically and apoA-I content was determined using the bicinchoninic acid (BCA) assay (Thermo-Fisher) with bovine serum albumin as standard.

### Lutein uptake by cultured ARPE-19 cells

ARPE-19 cells were cultured for 6 days in 100 mm culture dishes prior to a 72 h incubation with media only, or media containing lutein ND (8.8 μM as lutein). Following incubation, cells were harvested, centrifuged at 700 × g and washed 3 × with PBS to remove unincorporated lutein. Methanol (400 μl) containing an internal standard (fucoxanthin) was added to the cell pellet and, after 15 min, the sample was centrifuged at 14,000 × g for 5 min. The resulting supernatant was collected, filtered through a 0.22 μm nylon membrane and analyzed by reversed phase HPLC.

### Lutein analysis by HPLC

Methanol extracts of ARPE-19 cells were chromatographed on a Shimadzu Prominence HPLC fitted with a Kinetex 5 μm EVO C8 100 Å, 150 × 4.6 mm reversed phase column (Phenomenex) and a SecurityGuard ULTRA for EVO C8 guard column. Samples were separated using a gradient of [A] methanol and [B] 97% methanol/3% 1 M ammonium acetate as mobile phases at a flow rate = 1.0 ml/min (column temperature = 30°C). The gradient was as follows: [A] 75%, [B] 25%, 0.01 min; [A] 50%, [B] 50%, 1.0 min; [A] 30%, [B] 70%, 15.0 min; [A] 0%, [B] 100%, 24.0 min. An SPD-M20A photodiode array detector was used to monitor absorbance at 440 nm.

### Cell viability assays

ARPE-19 cells were seeded at 4,000 cells per 100 μl in a 96-well microtiter plate. On day 7, cells were treated with media only, media supplemented with empty ND or media supplemented with specified concentrations of lutein ND (0.9, 1.8, 3.5, and 8.8 μM as lutein) in PBS. Following 72 h incubation, the CellQuanti-Blue assay (BioAssay Systems) was used to measure cell viability. Sample fluorescence emission was measured at 590 nm (excitation 530 nm) on a Spectramax M5 plate reader.

### UV irradiation studies

Experiments were performed using a UVP Blak-Ray, Xx-15BLB, 115 V lamp (Analytik Jena, United States) elevated 10 cm above a 96-well plate containing ARPE-19 cells. ARPE-19 cells (4,000 cells per 100 μl) were cultured in a 96-well plate. On day 7, prior to UV irradiation, the culture media was removed and replaced with a minimal volume (30 μl; sufficient to cover the cells) of phenol red-deficient media. Plates were then exposed to UV-A (365 nm) irradiation for 2 h followed by determination of cell viability. In other experiments, ROS generation was measured using the DCDFA/H2DCDFA Cellular ROS Assay (Abcam, Cambridge, MA,United States), according to the manufacturer’s instructions.

### Statistical methods

Statistical analyses were performed by paired t-test (Figure 3) or two-way ANOVA followed by Dunnett’s multiple comparison test to compare treated samples against control samples (Figures 4–6). Samples treated with media alone served as negative control for Figures 4, 5 while samples treated with UV light served as positive control for Figure 6. Statistical tests were performed using GraphPad Prism version 9.1.1 for Windows (GraphPad Software, San Diego, CA).

## Results

### Lutein solubilization efficiency studies

The relative ability of different ND formulation components to affect lutein solubility in PBS was assessed in solubilization efficiency experiments (Table 1). Specified ND components were combined with lutein and bath sonicated. Following sonication, samples were centrifuged to remove insoluble material and lutein content in the supernatant fraction measured. In reaction mixtures containing PBS only, lutein was completely insoluble. In reaction mixtures containing lutein plus apoA-I or lutein plus EYPC, solubilization efficiencies of 19% and 37%, respectively, were observed. In reaction mixtures containing lutein, EYPC and apoA-I, solubilization efficiency was ~90%. Thus, when both a bilayer forming phospholipid and apolipoprotein scaffold protein are included in the formulation mixture, aqueous soluble lutein ND are produced.

### UV/Vis absorbance spectroscopy

**FIGURE 1:**
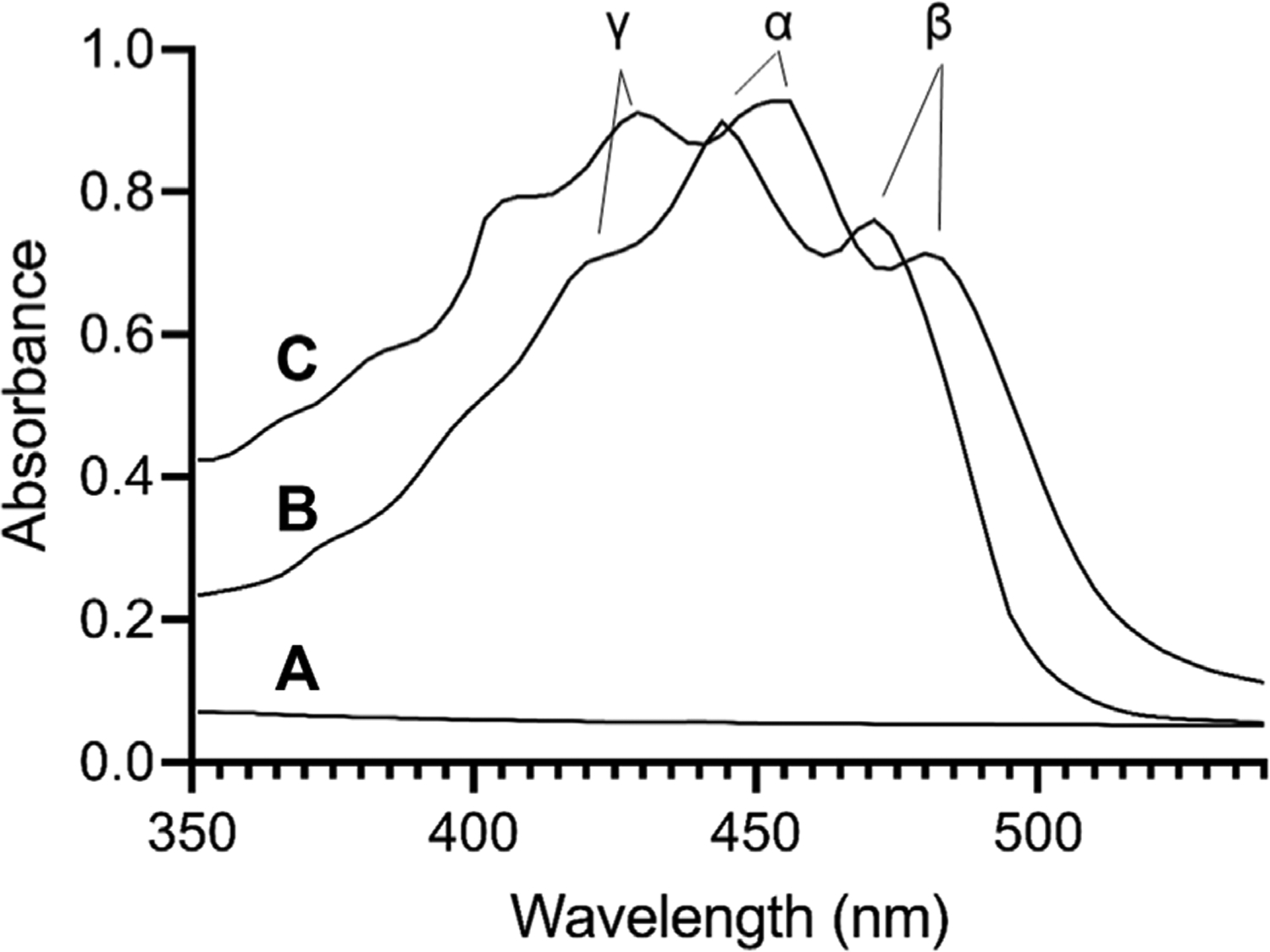
UV/Vis absorbance spectroscopy of lutein. Spectra were collected for (**A**) control EYPC rHDL (no lutein) in PBS; (**B**) lutein (10 μg) in ethanol; (**C**) lutein ND (10 μg as lutein) in PBS. Samples were scanned from 350 to 570 nm on a Spectramax M5 spectrophotometer.

To characterize the effect of ND incorporation on the spectral properties of lutein, UV/Vis absorbance spectra were collected. Spectra of free lutein (in ethanol) and lutein ND (in PBS) are presented in Figure 1. In ethanol, lutein gives rise to characteristic absorbance maxima at γ = 424, α = 445, and β = 472 nm. (Davies and Goodwin, 1976). In the case of control ND (no lutein), negligible absorbance was detected in this wavelength range. When spectra of lutein ND were recorded, a pattern similar to lutein in ethanol was detected. Unlike lutein in organic solvent, however, characteristic absorbance maxima were red shifted (γ = 430, α = 455, β = 485 nm). Thus, although free lutein in ethanol and lutein ND in PBS yield similar spectra (both display characteristic γ, α, and β peak maxima), the absorbance maxima of the γ, α, and β peaks in lutein ND undergo a 6–13 nm bathochromic shift. The red shift in lutein absorbance maxima for lutein ND in PBS versus free lutein in ethanol may be attributed to a change in environment (i.e., solvent polarity).

### FPLC gel filtration chromatography of lutein ND

**FIGURE 2:**
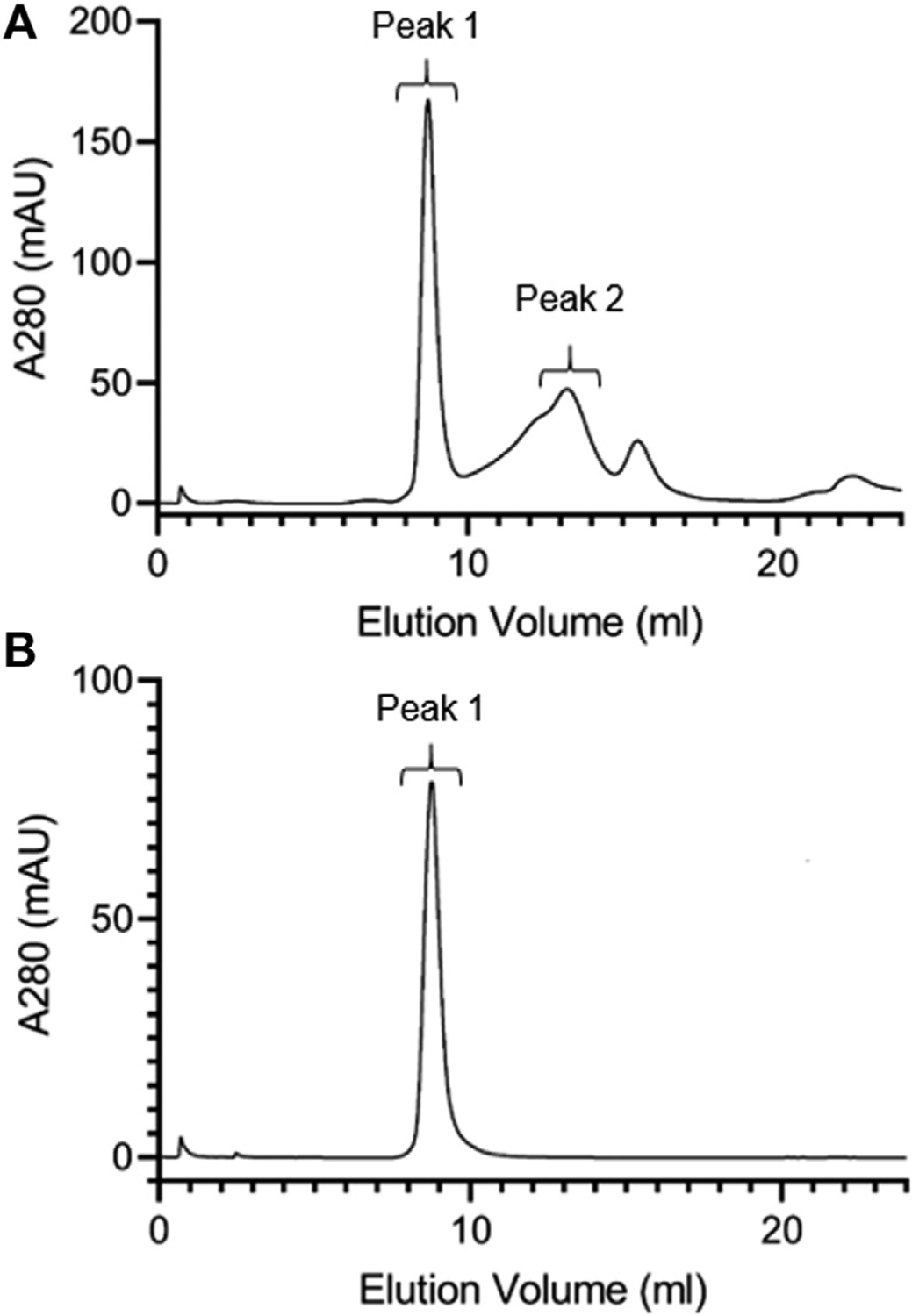
FPLC gel filtration chromatography of lutein ND. A 400 μl aliquot of freshly prepared lutein ND (corresponding to 0.8 mg apoA-I and 0.4 mg lutein) was analyzed by FPLC gel filtration chromatography, with absorbance continuously monitored at 280 nm (**A**). Fractions corresponding to Peak 1 from Panel a were pooled, concentrated and subjected to FPLC gel filtration analysis under the same conditions (**B**).

To characterize lutein ND particle size, FPLC gel filtration chromatography was performed (Figure 2). Chromatograms of freshly prepared lutein ND gave rise to a major 280 nm absorbance peak that elutes from 8.2 to 10.2 ml. In addition, a minor absorbance peak eluted between 12.2 and 14.2 ml. Fractions corresponding to these peaks were collected and analyzed for lutein and phospholipid. The major peak contained 99% of the lutein present in the original sample. Likewise, >99% of the phospholipid was recovered in the major absorbance peak. Based on this elution pattern, it may be concluded that the minor 280 nm absorbance peak corresponds to apoA-I not incorporated into ND while the major peak (8.2–10.2 ml elution volume) represents lutein ND. Comparison of this peak to protein standards suggests a particle size in the range of 200–300 kDa, similar to that observed previously. (Redmond et al., 2007). The result that nearly all lutein in the ND formulation mixture eluted in this peak indicates that lutein has been solubilized in ND. To verify this, the major FPLC peak was collected, concentrated and subjected to FPLC gel filtration chromatography (Figure 2B). The results obtained reveal a homogeneous population of ND particles.

### ND-mediated delivery of lutein to ARPE-19 cells

**FIGURE 3:**
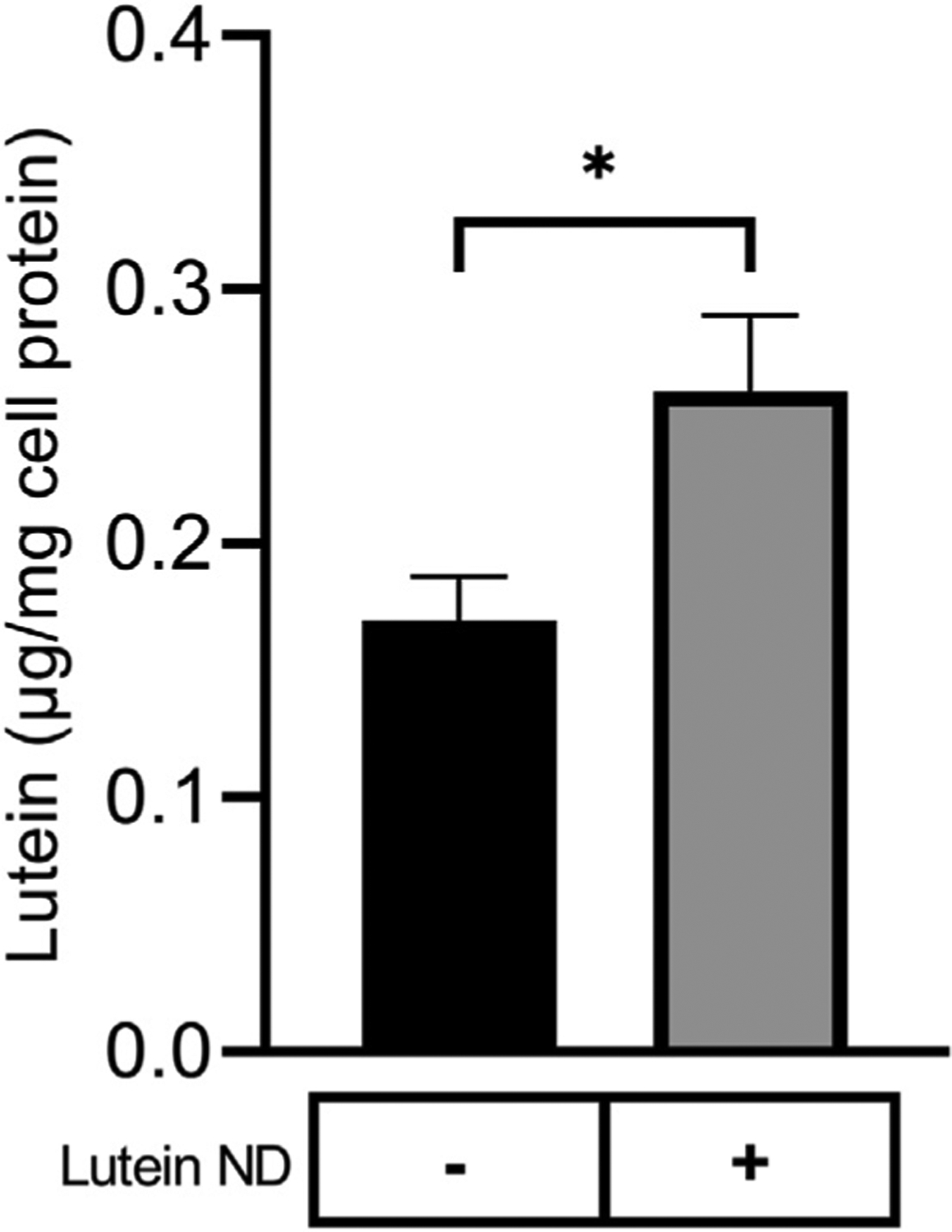
Lutein uptake by cultured ARPE-19 cells. ARPE-19 cells were incubated for 72 h in media only or media supplemented with lutein ND (8.8 μM as lutein). Following incubation, cells were washed to remove unincorporated lutein and suspended in methanol containing fucoxanthin as internal standard. The samples were homogenized, filtered and lutein content analyzed by reversed phase HPLC. Values reported are the mean ± standard error (*n* = 3); p = *p* < 0.05 vs. control.

To evaluate the ability of lutein ND to function as a delivery vehicle, ARPE-19 cells were incubated with media only or media containing lutein ND. Following incubation and washing, cells were extracted and analyzed for lutein (Figure 3). Compared to ARPE-19 cells incubated with media only, cells incubated with lutein ND (8.8 μM as lutein) possessed 35% ± 13.4% (*n* = 3) more lutein per mg cell protein.

### Effect of lutein uptake on ARPE-19 cell viability

**FIGURE 4:**
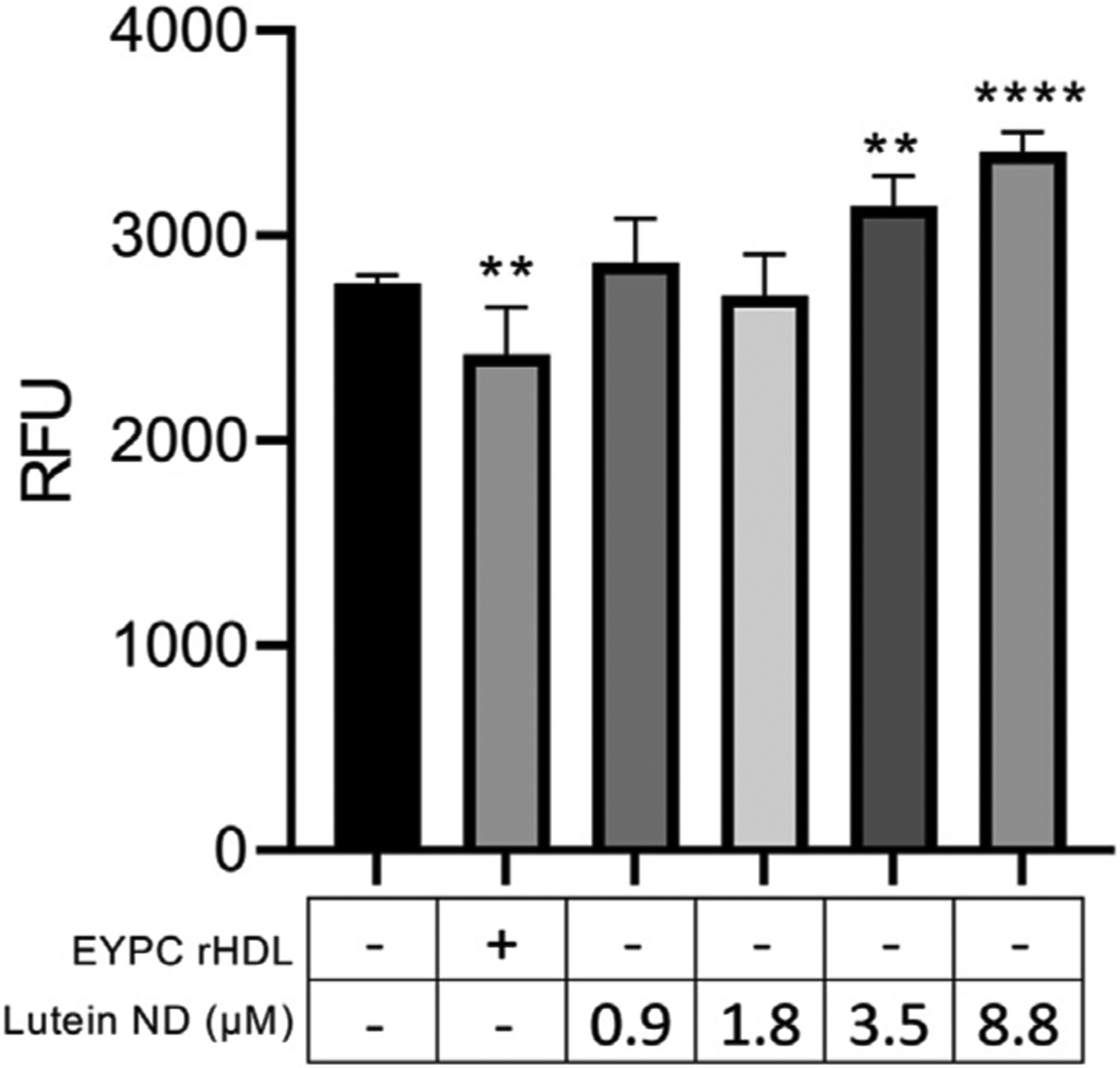
Effect of lutein ND on ARPE-19 cell viability. ARPE-19 cells were incubated for 72 h in media only, media supplemented with EYPC rHDL (no lutein), or media supplemented with indicated concentrations of lutein ND. Following incubation, cell viability assays were performed. Sample fluorescence emission was measured at 590 nm (excitation 530 nm). Values reported are the mean ± standard error (*n* = 6); pp = *p* < 0.01, pppp = *p* < 0.0001 vs. control.

ARPE-19 cells were incubated for 72 h with media alone, empty ND, or one of 4 concentrations of lutein ND. Subsequent cell viability measurements (Figure 4) revealed that, while cells incubated with empty ND showed a statistically significant reduction in cell viability, cells incubated with lutein ND displayed a lutein concentration-dependent increase in cell viability.

### Effect of UV irradiation on control and lutein-enriched ARPE-19 cells

**FIGURE 5:**
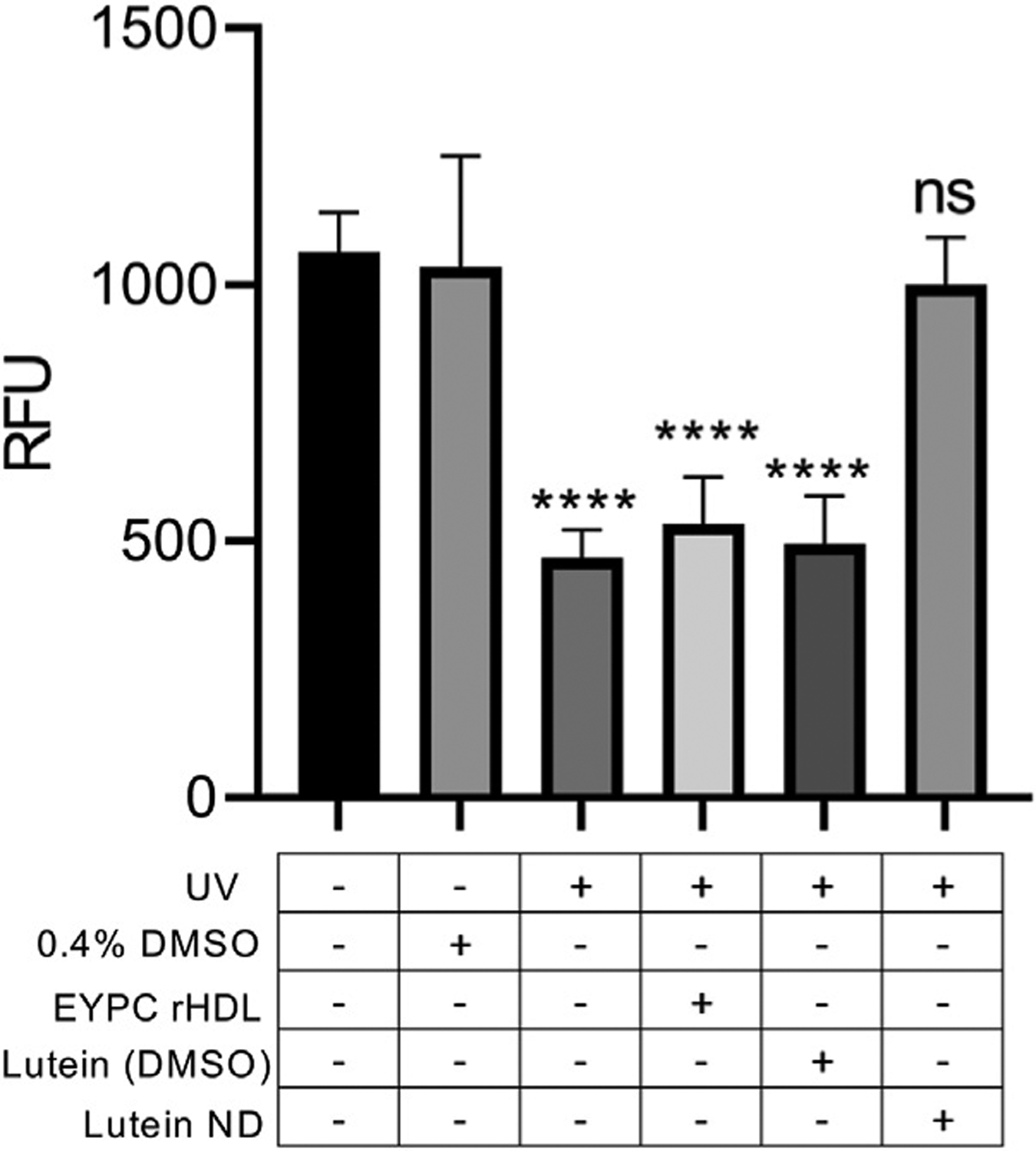
Effect of UV irradiation on ARPE-19 cell viability. Cells were incubated for 72 h in media only, media + 0.4% DMSO, media + EYPC rHDL, media + lutein (8.8 μM) in DMSO, or media + lutein ND (8.8 μM as lutein). Following incubation, cells in indicated treatment groups were exposed to UV irradiation for 2 h. Following irradiation, cell viability was assayed and sample fluorescence emission monitored at 590 nm (excitation 530 nm). Values reported are the mean ± standard error (*n* = 10); **** = *p* < 0.0001 vs. control, ns = not significant.

To determine the ability of ND-derived lutein to mitigate UV irradiation-induced damage, ARPE-19 cells were incubated for 72 h in the absence or presence of control EYPC rHDL, lutein in DMSO or lutein ND (in PBS). Following incubation, cells were washed and exposed to UV light for 2 h. Compared to control cells not exposed to UV light, 2 h exposure to UV irradiation resulted in an ~50% decline in cell viability (Figure 5). By contrast, UV irradiation of ARPE-19 cells following incubation with lutein ND displayed the same viability as cells not exposed to UV light.

### Effect of lutein ND on UV irradiation-induced ROS generation by ARPE-19 cells

**FIGURE 6:**
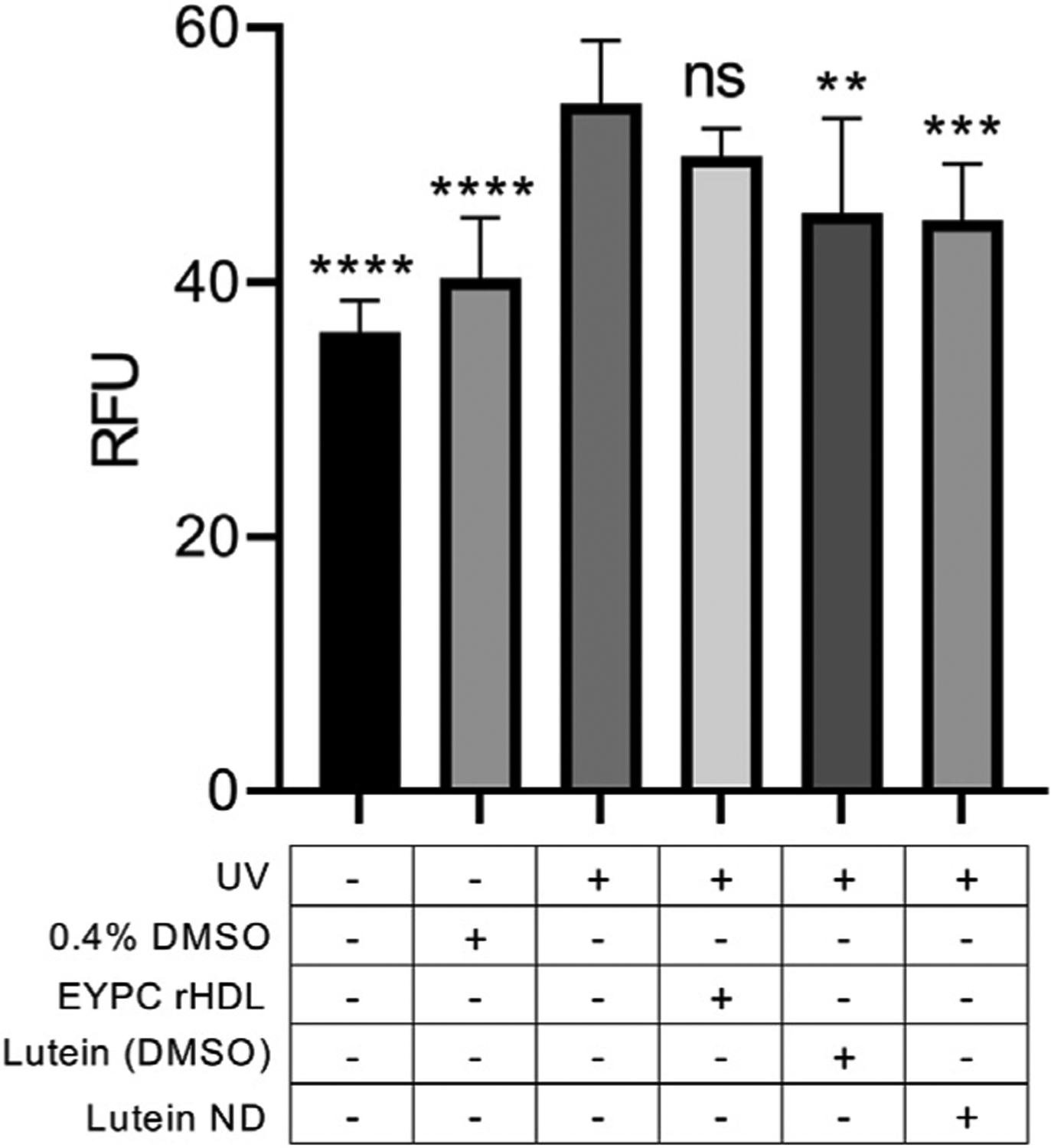
Effect of lutein ND on UV irradiation-induced ROS generation in cultured ARPE-19 cells. Cells were incubated for 72 h in media only, media plus 0.4% DMSO, EYPC rHDL, lutein (8.8 μM in DMSO), and lutein ND (8.8 μM as lutein). Following incubation, cells in indicated treatment groups were exposed to UV irradiation for 2 h. Following irradiation, cellular ROS levels were determined. For assay readout, sample fluorescence emission was measured at 535 nm (excitation 485 nm). Statistical values reported are the mean ± standard error (*n* = 10); ** = *p* < 0.01 vs. control, *** = *p* < 0.001 vs. control, **** = *p* < 0.0001 vs. control. Ns = not significant.

ARPE-19 cells were incubated in the presence or absence of lutein ND for 72 h followed by exposure to UV light for 2 h. Subsequently, cellular ROS levels were measured (Figure 6). Control ARPE-19 cells exposed to UV irradiation manifested an ~31% increase in ROS content compared to ARPE-19 cells not exposed to UV light. When cells were pre-incubated with lutein ND (8.8 μM as lutein), however, the UV light-induced increase in cellular ROS levels was 17%.

## Discussion

Lutein is an oxygenated carotenoid that serves essential functions in the eye as both an antioxidant and blue light filter. Because humans are incapable of synthesizing lutein, this highly hydrophobic xanthophyll must be obtained in the diet. The observation that eliminating lutein from the diet of experimental animals results in early signs of retinal degeneration (Kijlstra et al., 2012) suggests lutein should be designated a vitamin. Among more than 750 carotenoids in nature, only lutein, zeaxanthin and meso-zeaxanthin accumulate in the foveal region of the retina and, therefore, can be classified as macular pigments. (Arunkumar et al., 2018). Patients with macular telangiectasia, an acquired condition associated with redistribution of macular pigments, display a loss of central vision. (Choi et al., 2017). In healthy subjects, the ability of dietary lutein intake to reduce the risk of AMD remains controversial. This may be related to lutein bioavailability and the efficiency with which it is deposited in the macula, processes known to be affected by gene polymorphisms. (Meyers et al., 2013). In terms of function, however, it is clear that lutein serves an ocular protective role through its ability to filter blue light and function as an antioxidant. (Arunkumar et al., 2018).

**FIGURE 7:**
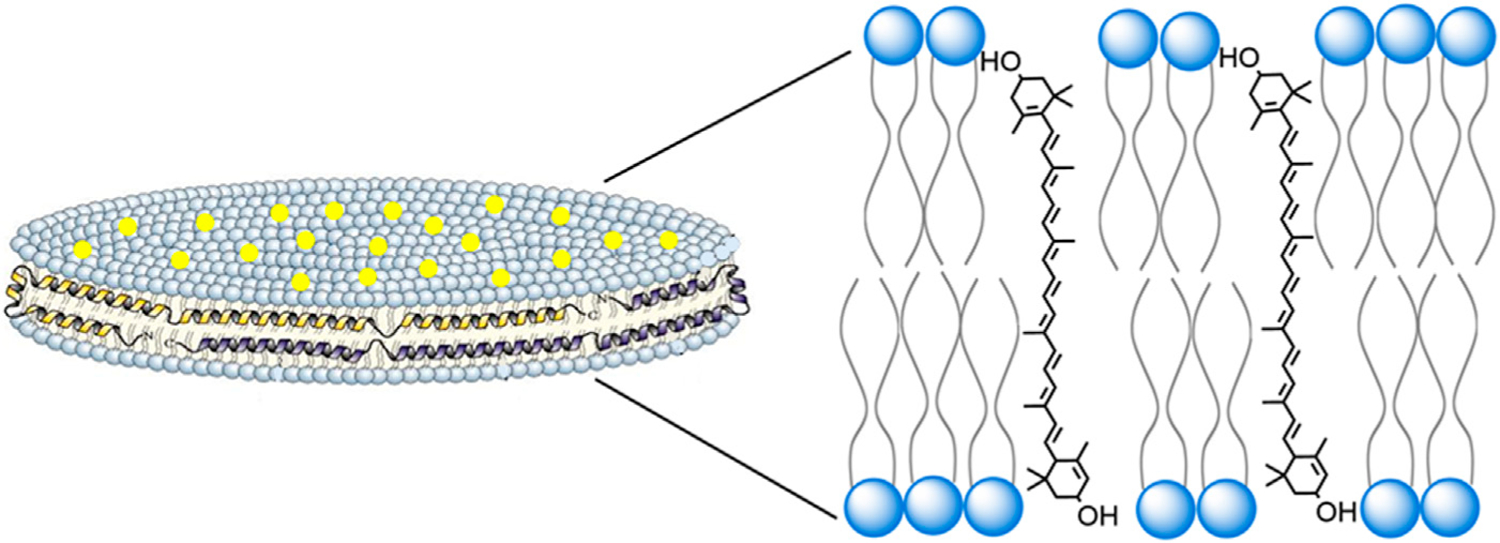
Structure and organization of lutein ND. A lutein ND particle is depicted showing a disk-shaped phospholipid bilayer with integrated lutein molecules (yellow dots). The disk is circumscribed by apolipoprotein scaffold molecules which contact otherwise solvent exposed phospholipid fatty acyl chains at the edge of the bilayer. In the expanded view, a plausible spatial orientation of lutein, with respect to the phospholipid bilayer, is presented. In this depiction lutein aligns parallel to phospholipid fatty acyl chains, such that its two hydroxyl moieties remain solvated, each localizing near phospholipid polar head groups at the aqueous interface.

Normally, lutein has very low aqueous solubility, in the range of 0.74 μg/ml (Drugbank online) Incorporation of lutein into ND, however, confers this xanthophyll with an ~1,300 fold increase in aqueous solubility (~0.9 mg/ml). Lutein ND contain only 3 components (phosphatidylcholine, apoA-I and lutein), organized as a discoidal lipid bilayer that is circumscribed by the amphipathic scaffold protein, apoA-I (Figure 7). In terms of biological activity, incubation of ARPE-19 cells with lutein ND led to an increase in cellular lutein content. When lutein ND-treated ARPE-19 cells were exposed to UV irradiation, they displayed improved cell viability and decreased ROS generation, as compared to control cells subjected to UV irradiation. These data indicate a direct photoprotective effect of lutein ND on these cells. The molecular basis underlying the protective effect of lutein on ARPE-19 cell oxidative damage has previously been investigated. For example, Kamoshita et al. (2016) observed that lutein treated ARPE-19 cells had increased expression and activity of superoxide dismutase (SOD) following light-induced oxidative stress. Consistent with increased SOD activity, these cells had reduced ROS levels. In a similar manner, Liu et al. (2017) reported that H_2_O_2_ treated ARPE-19 cells manifest a higher rate of cell cycle arrest versus untreated control cells. Importantly, however, pretreatment of these cells with lutein led to a concentration-dependent reduction in cell cycle arrest, as well as reduced ROS generation.

Uptake and transport studies have revealed that lutein associates with plasma lipoproteins. The consensus view is that lutein delivery to RPE cells occurs via HDL (Connor et al., 2007). The major protein component of HDL, apoA-I, serves as a ligand for scavenger receptor class B1 (SR-B1), which promotes selective uptake of lutein (Sato et al., 2013). RPE cells are located between the choroid vasculature and light absorbing rod and cone cells. Thus, as lutein is absorbed, it passes through this epithelial cell layer to reach its ultimate destination in stacked disks located in the outer segment of rod and cone cells. These disks, which exist as bilayer membrane sheets, are enriched in the light absorbing transmembrane protein, rhodopsin. Thus, outer segment disk structures serve as a platform upon which incident light impinges. Sequestration of lutein, and other macular pigments in these membranes protect phospholipids and protein from oxidation, preventing phototoxic damage.

The structural organization of cone and rod cell outer segment disks is similar to that of lutein ND in that both exist as phospholipid bilayers with embedded lutein. Thus, it is reasonable to consider that the orientation adopted by lutein in ND is similar to that in rod and cone outer segment disk membranes. Lutein is an oxygenated carotenoid (xanthophyll), possessing two hydroxyl moieties located at opposite ends of the molecule. Interestingly, the extended length of a lutein molecule approximates the width of a phospholipid bilayer. Studies of another hydrophobic membrane lipid, cholesterol, revealed that its single hydroxyl group orients between polar head groups of membrane phospholipids. (Kessel et al., 2001). Based on available evidence, it is plausible to consider that lutein behaves in a similar manner, aligning parallel, or at a tilted angle, relative to phospholipid fatty acyl chains in the membrane, while spanning the width of the bilayer (Figure 7). (Grudzinski et al., 2017) In this orientation, each lutein hydroxyl is positioned between phospholipid polar head groups on opposite leaflets of the bilayer.

Given the relative ease of ND formulation, lutein solubilization efficiency and loading capacity, lutein ND provide a means to bypass the digestive system through intravenous administration. This route is likely to enhance lutein delivery to the macula since it is administered directly to the bloodstream as a component of HDL mimetic nanoparticles that possess the SR-B1 ligand, apoA-I. It is noteworthy that rHDL particles comprised of phospholipid and apoA-I show promise as a treatment for atherosclerotic heart disease. (Kingwell et al., 2014). In human clinical trials, large quantities of rHDL have been administered intravenously without adverse effect. Given that rHDL are well tolerated, it is anticipated that lutein ND, which share a similar structure and component parts, will not manifest *in vivo* toxicity. Further study is required to evaluate the relative ability of diet-derived, versus intravenously administered lutein to reach the macula. It is further anticipated that ND can be formulated that possess more than one xanthophyll. By mimicking the relative proportions of macular pigments (lutein, zeaxanthin, and *meso*-zeaxanthin) in ND delivery particles, it may be possible to achieve a synergistic effect. If so, it is conceivable that mixed xanthophyll ND therapy could slow progression of AMD.

In conclusion, the present data illustrates that incorporation of lutein into ND dramatically increases lutein solubility. Furthermore, lutein ND-mediated delivery of lutein to cultured retinal pigment epithelial cells increases their lutein content. Given that the concentration of lutein in the macula naturally declines with age, (Obana et al., 2008), lutein ND represent a potential therapy option to slow or prevent progression of AMD in high risk individuals. In addition to intravenously administered lutein ND, it is conceivable that lutein ND could be administered ophthalmically, *via* eye drops. Although obstacles exist for this route, (Himawan et al., 2019), methods exist to determine whether topical administration of lutein ND will improve pigment deposition in the macula or slow progression of AMD. Thus, the potential for enhanced lutein bioavailability and increased macular accumulation through ND technology represents a promising strategy for prevention/delay of AMD.

## Figures and Tables

**TABLE 1:** Effect of ND formulation components on lutein solubility.

Component(s)^a^	Solubilization efficiency (%)
Lutein	<0.1
Lutein + apoA-I	18.8
Lutein + EYPC	37.4
Lutein + EYPC + apoA-I	89.7

a Indicated components were mixed with lutein (1 mg) and subjected to the formulation procedure described in Materials and Methods. Following bath sonication, the samples were centrifuged to remove insoluble material and the lutein content in the supernatant fraction determined by spectroscopic analysis.

b Solubilization efficiency (%) = the amount of lutein recovered in the supernatant fraction/total lutein added to the incubation × 100.

## Data Availability

The raw data supporting the conclusion of this article will be made available by the authors, without undue reservation.

## Abbreviations:

AMD: Age related macular degeneration
RPE: retinal pigment epithelium
ARPE-19: Spontaneously arising retinal pigment epithelial 19 cells
HDL: high density lipoprotein
rHDL: reconstituted high-density lipoprotein
ND: nanodisks
ROS: Reactive oxygen species
EYPC: Egg yolk phosphatidylcholine
ApoA-I: Apolipoprotein A-I
SR-B1: scavenger receptor class B type 1
FPLC: Fast protein liquid chromatography
